# Combination effects of tissue heterogeneity and geometric targeting error in stereotactic body radiotherapy for lung cancer using CyberKnife

**DOI:** 10.1120/jacmp.v16i5.5397

**Published:** 2015-09-08

**Authors:** Ki Mun Kang, Bae Kwon Jeong, Hoon‐Sik Choi, Seung Hoon Yoo, Ui‐Jung Hwang, Young Kyung Lim, Hojin Jeong

**Affiliations:** ^1^ Department of Radiation Oncology School of Medicine, Institute of Health Science, Gyeongsang National University JinJu Republic of Korea; ^2^ Department of Radiation Oncology Gyeongsang National University Hospital Jinju Republic of Korea; ^3^ Division of Heavy Ion Clinical Research Korea Institute of Radiological and Medical Science Seoul Republic of Korea; ^4^ Department of Radiation Oncology National Medical Center Seoul Republic of Korea; ^5^ Proton Therapy Center National Cancer Center Goyang Republic of Korea

**Keywords:** stereotactic body radiotherapy, SBRT, lung cancer, effective path length correction, Monte Carlo, tissue heterogeneity, geometric error

## Abstract

We have investigated the combined effect of tissue heterogeneity and its variation associated with geometric error in stereotactic body radiotherapy (SBRT) for lung cancer. The treatment plans for eight lung cancer patients were calculated using effective path length (EPL) correction and Monte Carlo (MC) algorithms, with both having the same beam configuration for each patient. These two kinds of plans for individual patients were then subsequently recalculated with adding systematic and random geometric errors. In the ordinary treatment plans calculated with no geometric offset, the EPL calculations, compared with the MC calculations, largely overestimated the doses to PTV by ∼21%, whereas the overestimation were markedly lower in GTV by ∼12% due to relatively higher density of GTV than of PTV. When recalculating the plans for individual patients with assigning the systematic and random geometric errors, no significant changes in the relative dose distribution, except for overall shift, were observed in the EPL calculations, whereas largely altered in the MC calculations with a consistent increase in dose to GTV. Considering the better accuracy of MC than EPL algorithms, the present results demonstrated the strong coupling of tissue heterogeneity and geometric error, thereby emphasizing the essential need for simultaneous correction for tissue heterogeneity and geometric targeting error in SBRT of lung cancer.

PACS numbers: 87.55.D, 87.55.kh, 87.53.Ly, 87.55.‐x

## I. INTRODUCTION

Lung cancer is the most common cancer and the leading cause of cancer‐related deaths worldwide as it accounts for approximately 12.7% of new cancers and 18.2% of cancer mortality.^(1)^ Treatment options for lung cancer include surgery,^(2)^ chemotherapy,^3^, ^4^ and radiotherapy (RT),^4^, ^5^ separately or in combination each other, but outcomes remain poor, even in patients with early‐stage lung cancer.^2^, ^3^, ^4^, ^5^ Stereotactic body radiotherapy (SBRT), which delivers an extremely hypofractionated dose to the tumor, has been raised as an alternative to conventional treatment options and has indeed shown promising results in several clinical trials.^6^, ^7^, ^8^

Two major issues must be addressed prior to the clinical implementation of SBRT for lung cancer: localization of movable tumor volume and correction for tissue heterogeneity. Localization of movable tumor volume is a geometric issue to concentrate a heavy radiation dose only to the target volume. Several techniques have been developed for this purpose, such as gating^9^, ^10^ and real‐time tumor tacking methods,^10^, ^11^, ^12^, ^13^ but none of these methods is completely precise. Thus, certain geometric margins, whether small or large, must be introduced for SBRT of lung cancer.^14^, ^15^

Correction for tissue heterogeneity is the issue related to the accuracy of dose calculation, which is particularly important when treating a tumor located in a strongly heterogeneous region such as a lung cancer.^16^, ^17^, ^18^ Three different correction algorithms are currently utilized for heterogeneity correction. The first is an empirical method, based on the effective path length (EPL) correction, using, for example, Batho's power law,^(19)^ (e.g., ray‐tracing^(20)^ and pencil beam algorithms^(21)^); the second is an analytical approximation for density‐dependent radiation transport (e.g., convolution superposition^(22)^); and the third is a Monte Carlo (MC) method,^18^, ^23^ which stochastically samples all possible physical interactions of radiation with tissue. Many studies have been performed to validate the dosimetric accuracies of these heterogeneity correction methods^23^, ^24^, ^25^ and have shown that the MC method is most accurate basically because it most comprehensively reflects the tissue heterogeneity effect.^24^, ^25^ In contrast, other algorithms, particularly the EPL‐based ray‐tracing and pencil beam algorithms, undercorrect for tissue heterogeneity effect, thereby overestimate the dose in the region of heterogeneity.^24^, ^25^, ^26^, ^27^, ^28^

These two issues for corrections of geometric targeting error and tissue heterogeneity have been separately addressed in conventional treatments by adding a geometric margin^14^, ^15^ and by using an appropriate heterogeneity correction method,^16^, ^17^, ^18^, ^19^, ^20^, ^21^, ^22^, ^23^, ^24^, ^25^, ^26^, ^27^, ^28^ respectively. However, these separate corrections may not be completely accurate because the targeting error can simultaneously alter the spatial tissue density distribution, thereby the dose distribution,^(29)^ suggesting a coupled effect of tissue heterogeneity and geometric targeting error. To date, however, little is known about the coupled effect. This study, therefore, quantitatively evaluated the coupled dosimetric effect in SBRT of lung cancer, using the fully heterogeneous MC and coarsely heterogeneous EPL functions incorporated in the CyberKnife planning system (MultiPlan ver. 3.5.4, Accuracy, Inc, Sunnyvale, CA).

## II. MATERIALS AND METHODS

### A. Treatment planning

This study utilized computed tomography (CT) scans taken of eight lung cancer patients. The grossly visible tumor volume (GTV) and critical structures, such as the spinal cord, lungs, esophagus, and heart, were delineated on the planning CT scan for each patient. The planning target volume (PTV) was expanded from the GTV by 3 mm to accommodate the targeting uncertainty during the treatment. The volumes of GTV and PTV ranged 1.7–33.9 cc and 4.5–52.5 cc, respectively. Tumors in the present patients were uniformly distributed in the upper, middle, and lower parts of both the right and left lungs (Table 1). The dose was equally prescribed to PTV for individual patients with a 60 Gy over 3 fractions.^(30)^

All the treatment plans for the individual patients were based on the CyberKnife SBRT technique with a real‐time tumor tracking method.^11^, ^12^, ^13^ The beams in CyberKnife are circularly collimated by using the 12 different sizes of fixed collimators (ranged 5 to 60 mm in diameter) or automatically variable “IRIS” collimator. Each four of eight patients were planned using the fixed and “IRIS” collimators, respectively, as given in Table 1

The plan optimization was performed only using the EPL algorithm with the following criteria: (i) the entire GTV and (ii) more than 95% of the PTV should receive the prescribed dose, (iii) 99% of the PTV should receive more than 95% of the prescribed dose, (iv) critical organs should receive lower doses than their recommended tolerances,^(31)^ and (v) the dose conformity index (CI), defined as the ratio of the PTV to the volume receiving more than the prescribed 60 Gy ($V_{60\text{Gy}}$), should be less than 1.2. For calculation efficiency, the optimization was performed in low resolution using a small calculation box that included only the volume of interests around the target volume. The prescription dose was basically normalized to ∼80% isodose line (range: 79%∼85%) with respect to the maximum dose point during the optimization step.

**Table 1:** Target volume characteristics and associated planning parameters used in this study

*Patient*	*Volume* ${\text{cm}}^{3}$		*No. of Beams*	*Total MUs*	*Collimator*		*Tx. Site*
	*GTV*	*PTV*			*Type*	*Size (mm)*	
A	1.7	4.5	186	30984	Fixed	10, 12.5, 15	RM
B	3.9	9.0	138	37171	IRIS	12.5, 15, 20, 25	RU
C	6.0	11.5	191	35587	Fixed	10, 12.5, 20	LL
D	10.7	18.9	151	36452	Fixed	10, 15, 25	RL
E	11.5	21.6	112	29718	IRIS	12.5, 15, 20, 25, 30, 35, 40	RU
F	27.6	36.8	143	30674	IRIS	12.5, 15, 20, 25, 30, 35, 40	LI
G	24.7	41.2	156	41611	Fixed	12.5, 25, 40	LI
H	33.9	52.5	192	35938	IRIS	12.5, 15, 20, 25, 30, 35, 40	LU

PTV = planning target volume; GTV = gross tumor volume; MUs = monitor units; Tx. Site = treatment site which is designated by two capital letters with the former representing the right (R) and left (L) lungs, respectively, and the latter representing the upper (U), middle (M), lower (L), and inguinal (I) lobes, respectively.

Once the optimization was completed, the final dose calculation was performed in high resolution on whole patient body to check for hot spot away from the target using both the EPL and MC algorithms. The contour correction scheme was used in the final dose calculation step to improve the radiological path length estimation. The MC plans were calculated with 1% relative statistical uncertainty (i.e., the dose deviation with historical sampling less than ∼1%) and were smoothened with a normalized Gaussian broadening width of 0.6. The recalculated EPL plans were equally normalized to have the same 95% of the PTV coverage. In contrast, the MC plans were either calculated with the same monitor units (MUs) (i.e., referred to as nonnormalized MC plan) or renormalized to have the same 95% PTV coverage with the corresponding EPL plans (i.e., referred to as renormalized MC plan). The target volumes and associated planning parameters are summarized in Table 1

### B. Dose difference between the MC and EPL plans with no geometric error

The dose differences between the EPL either nonnormalized or renormalized MC plans for individuals were primarily estimated by determining the doses delivered to 99% of the GTV and PTV, referred to as $D_{99\%}\;(\text{GTV})$ and $D_{99\%}\;(\text{PTV})$, respectively. In addition, the differences in doses to critical structures were estimated by the doses delivered to the hottest 1% of the structural volumes ($D_{1\%}$). Other dosimetric quantities, such as the mean doses ($D_{\text{mean}}$), the maximal ($D_{\text{max}}$) doses, and prescribed dose coverages ($V_{60\text{Gy}}$) for PTV and GTV, and the volume of the lung receiving at least 20 Gy ($V_{20\text{Gy}}$), were also estimated to analyze the dose difference between the EPL and MC plans (Table 2).

**Table 2:** Dosimetric data for the EPL and MC plans. The MC plans for individual patients were calculated with the same MUs (nonnormalized) either renormalized to have the same 95% PTV coverage with the corresponding EPL plans. All the doses and volumes, except for the prescription isodose line (Rx IDL), are given in percentage relative to the prescribed dose and the entire volumes of corresponding structures, respectively. The Rx IDL are given in percent ratio between the prescribed dose and the maximal doses in individual plans

*Structure*	*Quantity*	*EPL*	*MC*	
			*Nonnormalized*	*Renormalized*
GTV	$D_{99\%}$	105±2(103−109)	93±5(84−102)	107±3(104−112)
	$D_{\text{mean}}$	112±3(107−116)	103±3(98−107)	121±4(118−129)
	$D_{\max}$	123±3(117−126)	117±3(112−121)	137±6(130−147)
	$V_{60\text{Gy}}$	100.0±0.0	72.5±21.0(33.6−94.2)	100.0±0.0
PTV	$D_{99\%}$	97±1(95−98)	79±6(69−89)	91±1(89–93)
	$D_{\text{mean}}$	108±2(103−110)	97±4 (88–102))	115±2(112−118)
	$D_{\max}$	123±3(117−126)	117±3(112−121)	137±6(130−147)
		95.0±0.0	47.9±26.0(12.6−89.8)	95.0±0.0
	$V_{60\text{Gy}}$ CI	1.1±0.1(1.0−1.3)	1.0±0.0	1.2±0.1(1.2−1.3)
	Rx IDL	81±2(79−85)	86±2(83−89)	71±3(68−77)
Lung	$D_{1\%}$	96±17(63−112)	86±18(49−102)	98±18(65−116)
	$V_{20\text{Gy}}$	6.3±4.5(1.2−14.5)	5.9±4.3(1.0−13.7)	6.9±4.9(1.5−16.1)
Heart	$D_{1\%}$	14±13(5−43)	14±13(5−43)	15±14(6−49)
Spinal Cord	$D_{1\%}$	11±8(4−24)	11±8(4−23)	13±9(5−26)
Esophagus	$D_{1\%}$	13±8(5−29)	12±8(5−27)	14±9(6−31)

All data are given in mean ± SD. The values in the parentheses represent the ranges for the corresponding quantities.

### C. Dose change caused by systematic targeting errors

To investigate the coupled dosimetric effect of the geometric targeting error and the tissue heterogeneity, the dose distributions in an ordinary treatment plan were recalculated after adding a systematic offset to the beam targeting positions along the cranial‐to‐caudal direction. This was performed by programming the “AutoHotKey” script language, a free and open‐source software for macrocreation and automation for the Window operating system (http://www.autohotkey.com). The magnitude of the uniform shift or the systematic error ranged between −5 mm and 5 mm in 1 mm intervals, where the minus and plus signs indicate the shifts toward the caudal and cranial directions, respectively.

The change in target dose associated with the systematic offset was primarily estimated from the dose to delivered GTV, using $D_{99\%}\;(\text{GTV})$, because the GTV was defined here as the unique tumor volume intended for treatment, whereas the PTV was introduced solely for planning purposes to prevent underdosage to the intended GTV caused by geometric error. In this study, if $D_{99\%}\;(\text{GTV})$ was greater than 95% of the prescribed dose, it was regarded as acceptable dose to the intended GTV.^14^, ^15^

### D. Dose change caused by random error

The CyberKnife continuously tracks respiratory tumor motion during beam delivery, under the assumption that tumor always follows a regular motion trajectory detected by the in‐room patient imaging system.^11^, ^12^, ^13^ Actual tumor motion, however, can deviate from the regular motion trajectory, with the deviation occurring in any direction with any magnitude and at any instant of beam delivery, indicating that a real geometric error would be a random‐type error rather than a systematic error.^(12)^ To mimic this more realistic situation, the change in tumor dose associated with the random geometric error was also investigated using a typical patient case (Patient H). Three different series of random motion sets, each consisting of 192 computer‐generated random numbers which were uniformly distributed in the range between 0 and 1, were prepared to mimic the random targeting errors along all three translational directions for the 192 beams in the treatment plan for this patient. The magnitude of the random error along each of the three translational axes of cranial‐to‐caudal (CC), left‐to‐right (LR), and anterior‐to‐posterior (AP) axes, was described by the standard deviation (SD) of the error along the axes (σCC,σLR, and σAP, respectively) and the overall magnitude of the error ($\sigma_{tot}$) was defined as the root‐square sum of the standard deviations as

(1) $$\sigma_{tot}=\sqrt{\sigma_{CC}+\sigma_{LR}+\sigma_{AP}}.$$

Three generated random number sets were linearly scaled so that the overall magnitude of the error ($\sigma_{tot}$) became integers in mm, ranging from 1–9 mm. These rescaled random‐number sets were then sequentially added into the CC, LR, and AP components of the beam targeting positions, respectively, using the AutoHotKey macro programming. The assigned random motion sets were almost equally weighted along the three translational axes (i.e., $\sigma_{rCC}\approx \sigma_{LR}\approx \sigma_{AP}$). The change in dose caused by the random geometric error was calculated using both the EPL and MC algorithms and evaluated using a method similar to that for the systematic error.

## III. RESULTS

### A. Dose difference between EPL and nonnormalized MC plans

The EPL‐based treatment plans for individual patients were prepared to meet the planning criteria described above, as shown in Table 2. The PTV and GTV coverages (95% and 100%, respectively, in all the patients), the $D_{99\%}$ (range: 95%–98%) and CI (range: 1.0–1.3) of the PTV, and the dose‐volume limits for the critical structures fully or almost fully satisfied their criteria.

**Figure 1:**
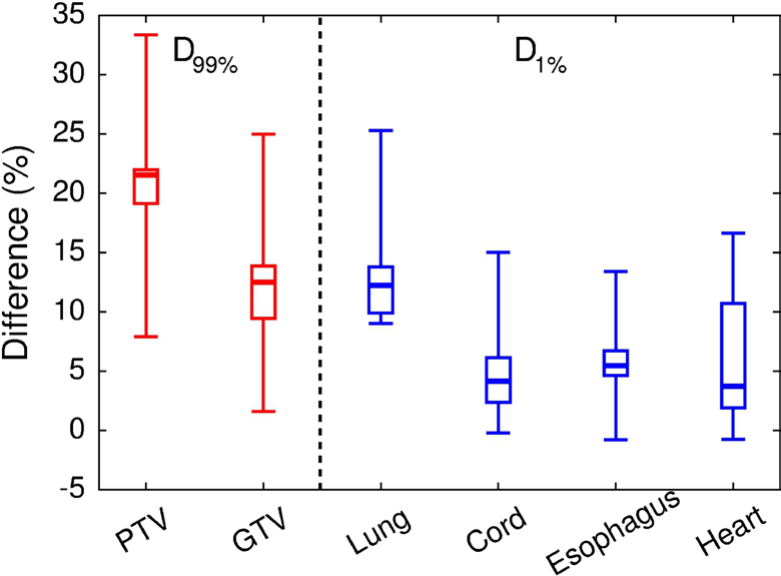
Box‐and‐whisker plots for differences in $D_{99\%}$ for tumor volumes (GTV and PTV) and $D_{1\%}$ for critical structures (lungs, spinal cord, esophagus, and heart) in between the EPL plans and MC plans recalculated with the same MUs. The differences are calculated as $2(D_{\text{EPL}}-D_{\text{MC}})/(D_{\text{EPL}}+D_{\text{MC}})$, where $D_{\text{EPL}}$ and $D_{\text{MC}}$ are the doses in the EPL and MC plans, respectively.

However, when recalculating the EPL plans using the MC algorithm with exactly the same MUs, the results were substantially deviated from the above criteria. The most significant deviation was the dramatic reduction in doses delivered to the target volumes. The prescribed dose coverages for PTV and GTV were decreased to 13%–89% from 95%, and to 34%–100% from 100.0%, respectively. The mean differences in $D_{99\%}$ of PTV and GTV between the EPL and MC plans for individual patients were 21%±7% and 12%±7%, respectively (Fig. 1 and Table 2). In our patients, the largest and second‐largest differences of GTV doses in between the EPL and MC plans were observed with Patient A (25%) with the smallest tumor volume and Patient H (18%) with the largest tumor volumes, respectively, and the smallest difference was observed with Patient F (2%) with medium‐sized tumor volume. These results demonstrated that there was no clear relationship between the dose difference and the tumor size (or the collimator size).

**Figure 2:**
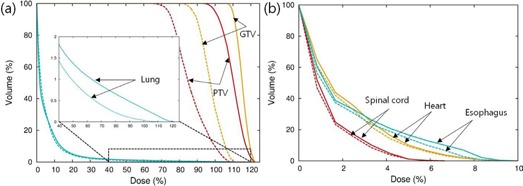
Dose‐volume histograms (DVHs) for Patient A calculated with the EPL (solid lines) and MC (dashed lines) algorithms, respectively: (a) DVHs for GTV, PTV, and lungs, and (b) DVHs for spinal cord, heart, and esophagus. The inset in (a) is the enlarged view of DVH for lungs.

The decreased doses resulting from the MC recalculation were also consistently observed in critical structures (Fig. 1 and Table 2). The mean differences in $D_{1\%}$ between the EPL and MC plans amounted to 5%±5% for the spinal cord, 6%±6% for the heart, and 6%±4% for the esophagus, which were much lower than the differences in doses delivered to tumor volumes. On the other hand, the mean difference in $D_{1\%}$ to lungs (12%±6%) was comparable to the difference in GTV dose between the EPL and MC plans (12%±7%). The overall difference in doses between the EPL and MC calculations is illustrated with the dose‐volume histograms (DVH) for Patient A, as shown in Fig. 2.

### B. Dose difference between EPL and renormalized MC plans with the same PTV coverage

The MC plans initially calculated with the same MUs as the corresponding EPL plans were linearly scaled to have the same 95% PTV coverage as the EPL plans. This linear scaling or renormalization substantially increased MUs, thereby increasing the doses in the MC plans. The overall magnitude of the increase was 14%±7% (range: 2%–28%), increasing $D_{99\%}\;(\text{GTV})$ and $D_{99\%}\;(\text{PTV})$ to 107%±3% (range: 104%–112%) and 91%±1% (range: 89%–93%), respectively. $D_{1\%}$ for other critical structures was also linearly increased with the renormalization, as given in Table 2

When evaluating the renormalized MC plans for individual patients based on the planning criteria used in the study, the requirements for PTV and GTV coverages (at least 95% and 100% coverages, respectively) were fully satisfied, but others were not (Table 2). For example, the $D_{99\%}\;(\text{PTV})$ in the renormalized MC plans, which ranged between 89%–93%, were smaller than the criterion of ≥95% in all our patients, and the CI for PTV (range: 1.2–1.3) exceeded the tolerance limit (≤1.2) in four of eight patients.

### C. Dose change with systematic error

**Figure 3:**
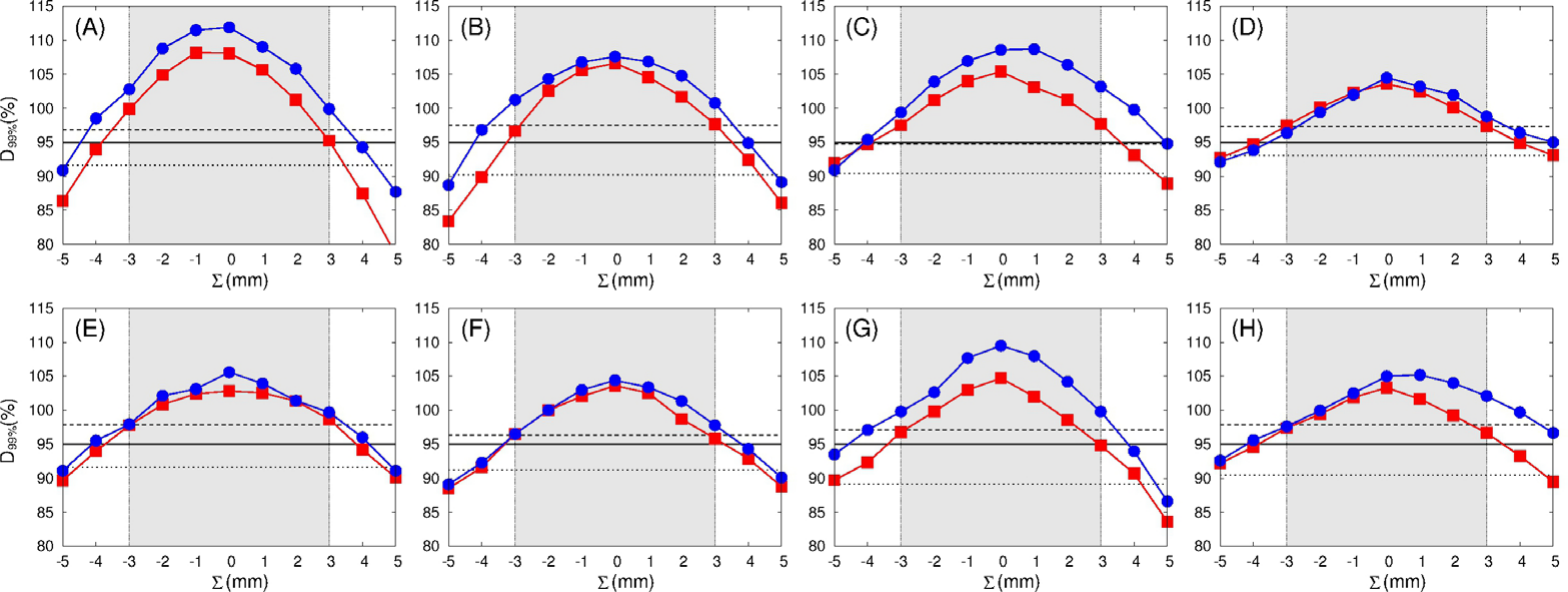
Variations of $D_{99\%}\;(\text{GTV})$ in the EPL (filled squares) and MC (filled circles) plans for individual patients (A to H) as a function of systematic beam offset (Σ) along the cranial‐to‐caudal direction. The plus (minus) sign for Σ indicates the shift toward the cranial (caudal) direction. The 95% prescription dose level (solid line), as well as the dose to 99% of PTV levels in the ordinary EPL (dashed line) and the renormalized MC (dotted line) plans, are given in each plot, for reference. The offset range less than the PTV margin (i.e., −3 mm Σ≤3 mm) is gray shaded in each figure.

**Figure 4:**
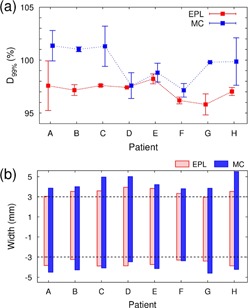
(a) Doses to 99% of GTV (i.e., $D_{99\%}\;(\text{GTV})$) calculated at ±3 mm offsets to craniocaudal direction using the EPL (red squares and error bars) and MC (blue squares and error bars) plans for individual patients (A to H), where the mean values between $D_{99\%}\;(\text{GTV})$ at +3 mm (toward the cranial) and −3 mm (toward the caudal direction) are marked by squares and the ranges of the values are indicated by error bars. (b) Acceptable ranges for systematic shift that resulted in $D_{99\%}(\text{GTV})\;\geq 95\%$ in EPL (red boxes) and MC (blue boxes) plans for individual patients, which were obtained with the interpolations of raw data given in Fig. 3.

**Figure 5:**
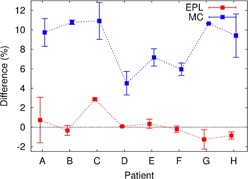
Differences between $D_{99\%}\;(\text{GTV})$ at ±3 mm offsets and the ordinary planned $D_{99\%}\;(\text{PTV})$ calculated using the EPL (red squares and error bars) and MC (blue squares and error bars) plans for individual patients (A to H). The mean of the differences between at +3 mm (toward the cranial) and −3 mm (toward the caudal direction) are marked by squares, and the minimal and maximal values of the differences are indicated by error bars.

The changes in $D_{99\%}\;(\text{GTV})$ with the systematic geometric offset (Σ) added along the CC axis are displayed in Fig. 3, where all the MC plans were renormalized to have a 95% PTV coverage before adding the geometric error. The GTV for individual patients consistently received higher doses in the MC than in the EPL plans when applying the systematic error. For example in examining Fig. 4(a), the $D_{99\%}\;(\text{GTV})$ calculated at ±3 mm offset in individual patients was 97%±1% in the EPL plans and 10%±2% in the MC plans, or ∼3% higher for the MC than the EPL plans. The relatively higher GTV doses in MC than in EPL calculations also can be seen in Fig. 4(b), where the range in geometric errors that result in clinically acceptable doses to the GTV (i.e., $D_{99\%}(\text{GTV})\;\geq 95\%$ was shown. The range for individual patients was consistently broader in the MC than in the EPL plans with the former having broader widths by 0.4±0.2 mm and 0.9±0.3 mm to caudal and cranial directions, respectively. The differences between $D_{99\%}\;(\text{GTV})$ recalculated with ±3 mm offsets and the ordinarily planned $D_{99\%}\;(\text{PTV})$ without geometric error are plotted in Fig. 5. The differences ranged only −2%–3% (average 0%±2%) in the EPL plans, but considerably increased to 3%–13% (average 9%±3%) in the MC plans.

### D. Dose changes with random errors

**Figure 6:**
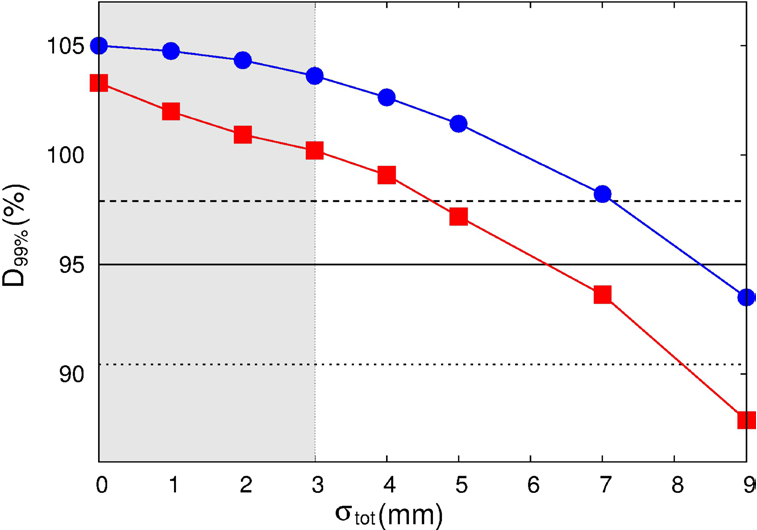
Variations of $D_{99\%}(\text{GTV})$ in the EPL (filled squares) and MC (filled circles) plans for Patient H as a function of random targeting error ($\sigma_{\text{tot}}$), where the 95% prescription dose level (solid line) as well as $D_{99\%}\;(\text{PTV})$ levels in the ordinary EPL (dashed line) and the renormalized MC (dotted lines) plans are given, for reference. The gay shaded region shows the error range less than the PTV margin (i.e., $-3\;\text{mm}\;\leq \sigma_{\text{tot}}\leq 3\;\text{mm}$).

The dose variations associated with the random geometric error displayed in Fig. 6 showed as similar trend to the variations resulting from the systematic error (3, 6). The $D_{99\%}\;(\text{GTV})$ was always higher in the MC than in the EPL calculations by 4%±1%, with the random geometric errors ranging 1–9 mm. However, these variations were smaller when compared to those caused by the systematic errors. For example, $D_{99\%}\;(\text{GTV})$ calculated at random errors of 1 mm, 3 mm, and 5 mm were smaller than those calculated with the same magnitudes of systematic errors, by 0%, 3%, and 6%, respectively, in the EPL calculations and by 1%, 4%, and 7%, respectively, in the MC calculations. The magnitudes of systematic errors that yielded the same $D_{99\%}\;(\text{GTV})$ values as those calculated with the random errors were estimated by interpolating the dose variation curves obtained with the systematic error shown in Fig. 3(h). The average ratios between the magnitudes of random and systematic errors having the same $D_{99\%}\;(\text{GTV})$ values were 1.6 in EPL and 1.9 in MC calculations.

## IV. DISCUSSION

This study investigated the dose distributions in SBRT treatment plans for lung cancer patients, as well as their variations associated with the tissue heterogeneity and geometric error, using the coarsely heterogeneous EPL and fully heterogeneous MC algorithms.

Compared with the MC dose, the EPL dose was consistently overestimated over all structures, although the magnitude varied among different structures. When evaluating the dose difference based on the $D_{1\%}$ to each structure, EPL dose was largely overestimated to lungs, a low‐density structure with water‐equivalent density ($\rho_{w}$) of 0.3±0.18, by ∼12%, but the overestimation was markedly lower in the relatively denser structures, such as the heart ($\rho_{w}=1.1\pm 0.03$), spinal cord ($\rho_{w}=1.0\pm 0.02$), and esophagus ($\rho_{w}=1.0\pm 0.15$), by ∼5%−7%, respectively, suggesting that change in dose due to the tissue heterogeneity effect was greater in lower density structure. Similarly, overestimation of the EPL dose to the relatively low‐density PTV ($\rho_{w}=0.4\pm 0.18$ for PTV periphery) was greater than that to the relatively denser GTV ($\rho_{w}=10\pm 012$). Among our patients, the differences between the EPL and MC calculations was amounted to 21%±7.3% for the $D_{99\%}\;(\text{PTV})$ and 12%±6.7% for the $D_{99\%}\;(\text{GTV})$. Similar findings for the tissue density dependence on dose in heterogeneous calculation(^16‐18^) and for the larger variation in dose to the PTV than to the GTV during SBRT for lung cancer(^26‐28^) have been observed in the previous studies.

In order to quantify the coupled dosimetric effect of tissue heterogeneity and geometric targeting error, we systematically investigated the change in dose distribution the change in dose distribution by applying the geometric error to the EPL and renormalized MC plans for individual patients. In the EPL calculation, systematic geometric error caused no significant change, except for overall shift, in the relative dose distribution. This was confirmed by our finding that the peripheral GTV dose (i.e., $D_{99\%}\;(\text{GTV})$) recalculated with the same magnitude of geometric offset as the PTV margin (here, 3 mm) agreed well with the ordinarily planned $D_{99\%}\;(\text{PTV})$, within 0%±1.6% (Fig. 5).

This situation, however, was markedly altered in the fully heterogeneous MC calculation, where the $D_{99\%}\;(\text{GTV})$ value calculated at 3 mm offset was significantly increased than the ordinarily planned $D_{99\%}\;(\text{PTV})$, as shown in Fig. 4 (average difference in our patients was 9%±3%). In addition, the $D_{99\%}\;(\text{GTV})$ recalculated with the systematic error was consistently higher in MC than EPL calculations (Fig. 2), even though the planned $D_{99\%}\;(\text{PTV})$ was rather consistently lower in the MC than EPL plans (Table 2).

The above difference between EPL and MC calculations can be explained in terms of the tissue density dependency between the EPL and MC algorithms.^16^, ^17^, ^18^ The EPL algorithm is not dependent on the spatial tissue density distribution, except for the line integrated tissue density along the primary beam axis, because it only corrects for the radiologic path length change of primary photon beam.^16^, ^17^, ^18^, ^24^, ^25^, ^26^, ^27^, ^28^ Although the geometric error can alter the radiologic path length or the line integrated tissue density along the beam axis, this effect may not be great, because the change in the radiologic path length may have the same order of magnitude as the geometric error, and the attenuation of megavoltage X‐ray beam across a few mm distance is normally very small.

In sharply contrast to EPL, MC has a strong dependency on the spatial tissue density distribution because MC simultaneously corrects for the transmittance dosage by lateral radiation scatterings, as well as the primary photon‐beam attenuation.^24^, ^25^ Because the transmittance dose is decreased with increasing the tissue density, the denser structure receives the higher dose in the MC calculation even when exposed by the same intensity of radiations. This suggests that the relatively denser gross tumor may receive higher dose than the planned dose to PTV when beams are mistargeted or the gross tumor is misplaced within the PTV margin range, and therefore the dose actually delivered to gross tumor cannot be estimated from the planned dose to PTV.

The similar change in dose to GTV was also observed with the random geometric error, although the change was significantly lower than that with the systematic error due to the blurring of dose distribution (∼1.9% times smaller in our MC calculation, as shown in Fig. 5).^(32)^ In addition, although not investigated here, the respiration‐induced tissue deformation can also alter the tissue density distribution. The effect of tissue deformation on tumor dosage in SBRT of lung cancer can be estimated by comparing the doses in between the three‐dimensional (3D) and the four‐dimensional (4D) plans. The difference was reported as ∼3% in a previous study^(28)^ which was smaller than the present difference induced by tissue density variation associated with geometric error (12%±7% for GTV dose difference, Table 2), suggesting the higher impact of the tissue density variation caused by geometric error than the respiratory‐induced tissue deformation on tumor dosage in SBRT of lung cancer.

The present findings demonstrated that tissue density variation associated with geometric errors should be carefully accounted for during the planning stage of SBRT for lung cancer. If assuming a static heterogeneity, it can be easily corrected by using an appropriate heterogeneous algorithm (e.g., MC). In contrast, changes due to geometric targeting errors or dynamic heterogeneity rather should be corrected by non‐ or less‐density–dependent algorithm, because the density variation cannot be predicted in a planning stage. This conflict for corrections of tissue heterogeneity and geometric error could be resolved by sequentially combining the coarsely heterogeneous EPL and fully heterogeneous MC algorithms. In this procedure, the density variation associated with geometric error is first corrected with the EPL algorithm by optimizing the plan so that the entire PTV receives the similar intensity of radiation or photon fluence, irrespective of its spatial tissue density distribution. This enables to deliver a similar intensity of radiations, thereby similar adsorbed dose, to the entire gross tumor, wherever sited within the range of planning margin. The tissue heterogeneity effect is then reflected by the following MC calculation, resulting in the successful corrections for both the dynamic and static tissue heterogeneity effects. The similar result can be found in the work by Lacornerie et al.,^(33)^ where they showed that the combination of using a EPL and MC algorithms could provide a robust method for treating heterogeneous lung lesions.

However, the procedure mentioned above requires a proper renormalization of the final heterogeneous MC dose because the required MU for delivery of prescribed dose is markedly underestimated in the EPL optimization step due to lack of consideration for transmittance dosage.^26^, ^27^, ^28^ Conventional recommendation for RT dose normalization includes (i) that at least 95% of the prescribed dose be delivered to the (near‐) entire PTV, and (ii) that 100% of the prescribed dose be delivered to at least 95% of the PTV.^14^, ^15^ These requirements basically arose from the simple assumption that the relative dose distribution in RT may not be significantly changed with the density variation caused by targeting error or tumor motion. This assumption, while reasonable for homogeneous situations, is not adequate for heterogeneous lung cancer since, for lung cancer, the dose distribution is sensitively changed depending on the spatial tissue density distribution. The former requirement was particularly excessive because the denser gross tumor generally receives much higher dose than the planned dose to PTV when it misplaced within range of the planning margin. The latter requirement (normalization to 95% of the PTV) alone was sufficient in our patients, as shown in Fig. 3, where the clinically acceptable dose (>95%) was delivered to the entire GTV even though the GTV was deviated from the planned position by the planning margin or the maximum allowed error for treatment.

The present method combined with the EPL and MC algorithms, though effective in simultaneous correction for static and dynamic tissue heterogeneities, has a limitation related to the fact that the final MC dose distribution is sometimes significantly deviated from the dose distribution previously optimized by the EPL algorithm. Therefore, it would be necessary to further improve the optimization efficiency without increasing the dosimetric uncertainty for SBRT planning for lung cancer. The MC‐based optimization, though the MC algorithm itself is obviously limited in correction for tissue density variation, could provide the solution for the improvement if it combined with the density overriding scheme (i.e., manual density override of PTV to the gross tumor density). This would be worthwhile to be investigated in a future work. In addition, though a 3 mm planning margin was only considered here based on the clinical protocol in our institution, it might be necessary to apply the present method to the different margin recipes (e.g., 5–8 mm PTV margin) in order to establish a clear relationship between geometric error and the actual dose delivered to GTV during SBRT of lung cancer.

## V. CONCLUSIONS

The present investigation quantitatively showed that not only tissue heterogeneity, but its variation associated with geometric error, considerably influences to the tumor dosage and, therefore, both the effects are carefully corrected in the planning stage of SBRT for lung cancer. These two effects can be simultaneously corrected by sequentially combining the EPL and MC algorithms to the dose optimization and final dose calculation steps, respectively. However, even after correcting for the effects, the planned PTV dose in the final heterogeneous MC calculation should be properly renormalized to deliver the sufficient dose to the gross tumor. An important point that should be considered is that the gross tumor, due to its higher density than surrounding normal lung tissues, receives higher dose than the planned dose to PTV, when it moves within the ranges of planning margin. Thus, weaker standards for dose normalization may be allowable in SBRT of lung cancer than in conventional treatments. The dose prescription to 95% of PTV was found to be sufficient in the present MC‐based planning for SBRT of lung cancer with a 3 mm planning margin. No additional requirements, such as dose to the entire or near‐entire PTV, were needed when the plan was optimized by the EPL algorithm prior to the heterogeneous MC calculation.

## ACKNOWLEDGMENTS

This Work (RPP‐2011‐021) was supported by the fund of Research Promotion Program, Gyeongsang National University, 2011.
